# Phage-antibiotic combinations to control *Pseudomonas aeruginosa*–*Candida* two-species biofilms

**DOI:** 10.1038/s41598-024-59444-2

**Published:** 2024-04-23

**Authors:** Prasanth Manohar, Belinda Loh, Ramesh Nachimuthu, Sebastian Leptihn

**Affiliations:** 1 School of Bioscience and Technology, Vellore Institute of Technology (VIT), Vellore, India; 2 Department of Vaccines and Infection Models, Fraunhofer Institute for Cell Therapy and Immunology (IZI), Perlickstr. 1, 04103 Leipzig, Germany; 3 Department of Biochemistry, Health and Medical University, Erfurt, Anger 66/73, 99084 Erfurt, Germany; 4 Department of Biochemistry and Molecular Biology, University of Southern Denmark, Odense, Denmark; 5 Present Address: Center for Phage Technology, Department of Biochemistry and Biophysics, Texas A&M AgriLife Research, Texas A&M University, College Station, TX 77843 USA

**Keywords:** Anti-biofilm activity, Phage therapy, Phage-antibiotic synergy, Polymicrobial infections, Dual-species biofilms, Extracellular matrix, Bacteriophages, Clinical microbiology

## Abstract

Phage-antibiotic combinations to treat bacterial infections are gaining increased attention due to the synergistic effects often observed when applying both components together. Most studies however focus on a single pathogen, although in many clinical cases multiple species are present at the site of infection. The aim of this study was to investigate the anti-biofilm activity of phage-antibiotic/antifungal combinations on single- and dual-species biofilms formed by *P. aeruginosa* and the fungal pathogen *Candida albicans*. The *Pseudomonas* phage Motto in combination with ciprofloxacin had significant anti-biofilm activity. We then compared biofilms formed by *P. aeruginosa* alone with the dual-species biofilms formed by bacteria and *C. albicans*. Here, we found that the phage together with the antifungal fluconazole was active against 6-h-old dual-species biofilms but showed only negligible activity against 24-h-old biofilms. This study lays the first foundation for potential therapeutic approaches to treat co-infections caused by bacteria and fungi using phage-antibiotic combinations.

## Introduction

Phage therapy makes use of bacteriophages to treat bacterial infections, in particular infectious ones that cannot be treated with conventional antibiotics due to multi-drug resistance^1,2^. Although most of the therapeutic applications of phages are in monotherapy or as phage cocktails, recently the combination of phages and antibiotics is getting attention through successful in vitro studies^3,4^. In most cases, phages and antibiotics were proven to act in synergy against bacteria^5–7^. Recent investigations showed that antibiotic resensitization can occur during phage exposure in antibiotic-resistant bacterial cells^8,9^.

*Pseudomonas aeruginosa* is a Gram-negative pathogen known to cause mild, but also life-threatening infections of the blood, the lungs and other parts of the body; here in particular after surgery. While many infections are treatable, an increasing number of antibiotic-resistant *P. aeruginosa* strains are being observed. This, together with the pathogen’s ability to form biofilms, can make the clinical control of infections challenging^10^. Recent studies showed the efficacy of *Pseudomonas* phages as anti-biofilm agents, while the use of phage-antibiotic combinations is gaining attention due to the noted synergistic effects^11–14^. Another major challenge with *P. aeruginosa* biofilms is that they often form as dual-species or multi-species biofilms^13,15^.

Microbe-microbe interactions can influence various infection parameters including virulence and biofilm formation. Dual-species biofilms formed by *P. aeruginosa* and *Candida albicans* are frequently found in cystic fibrosis patients but also in patients in association with catheters^15,16^. *Candida albicans* alone can cause opportunistic infections and is mostly found in the gastrointestinal tract, mouth and vagina^17^. Most bacterial-fungal dual-species biofilm infections are caused by the coexistence of *Pseudomonas*-*Candida* or *Staphylococcus*-*Candida*^15,17^. It is also known that the presence of *P. aeruginosa* can influence the *C. albicans* biofilms^18^ and longer exposure can inhibit the dual-species biofilm growth under in vitro conditions^19,20^. To date, antibiotics are the physicians’ choice for the treatment against dual-species biofilm infections but the recently increasing rates of antibiotic resistance can make the therapeutic approach unsuccessful and lead to life-threatening complications.

To understand the potential of using phage-antibiotic combinations on *Pseudomonas*-*Candida* embedded in biofilms, we tested various antibiotics and fungicides on single and dual-species biofilms. First, we systematically tested the effects of phages together with antibiotics on planktonic and then on biofilm-embedded bacteria before we studied the impact of antimicrobials in conjunction with phages on *Pseudomonas*-*Candida* biofilms*.* The hypothesis is that the biofilms formed by the *Pseudomonas-Candida* have exopolysaccharides which can be degraded by phage depolymerases so that the disturbed biofilm cells can be eliminated by phage Motto (as an anti-bacterial) and fluconazole (as an anti-fungal). The phage Motto used in this study was previously characterized to have anti-biofilm activity and found to have broad host range infectivity^21,22^. The anti-fungal fluconazole inhibits the fungal cell membrane synthesis and is mainly used to treat candidiasis and it has weak anti-biofilm activity.

## Results

### Antibiotics act synergistically with phage on planktonic cells

Phage-antibiotic combinations have attracted attention in the treatment of multidrug-resistant bacterial infections due to often observed synergistic effects in vitro and in vivo. We thus aimed to test the effect of phage Motto on its host in the presence of antibiotics. First, we determined the MIC of *P. aeruginosa* PA01 against different antibiotics. Five antibiotics, cefotaxime, ciprofloxacin, gentamicin, meropenem and tetracycline, were used in this study, which are either front-line or broad-spectrum antibiotics against *P. aeruginosa* infections. The isolate was found to be resistant to cefotaxime, gentamicin, meropenem and tetracycline but susceptible to ciprofloxacin according to the CLSI guidelines (Table 1).

**Table 1:** Representation of the minimal inhibitory concentration (MIC) results of *P. aeruginosa* PA01 strain and the sub-inhibitory concentrations chosen for the study.

S.no	Antibiotics	MIC (µg/mL)	1/4th sub-inhibitory MIC (µg/mL)
1	Cefotaxime	**64**	16
2	Ciprofloxacin	2	0.5
3	Gentamicin	**16**	4
4	Meropenem	**32**	8
5	Tetracycline	**32**	8

The resistance breakpoint (µg/mL) of Cefotaxime is ≥ 64, Ciprofloxacin is ≥ 4, Gentamicin is ≥ 16, Meropenem is ≥ 16, Tetracycline is ≥ 16 according to CLSI guidelines. The numbers in bold represent resistance.

**Figure 1:**
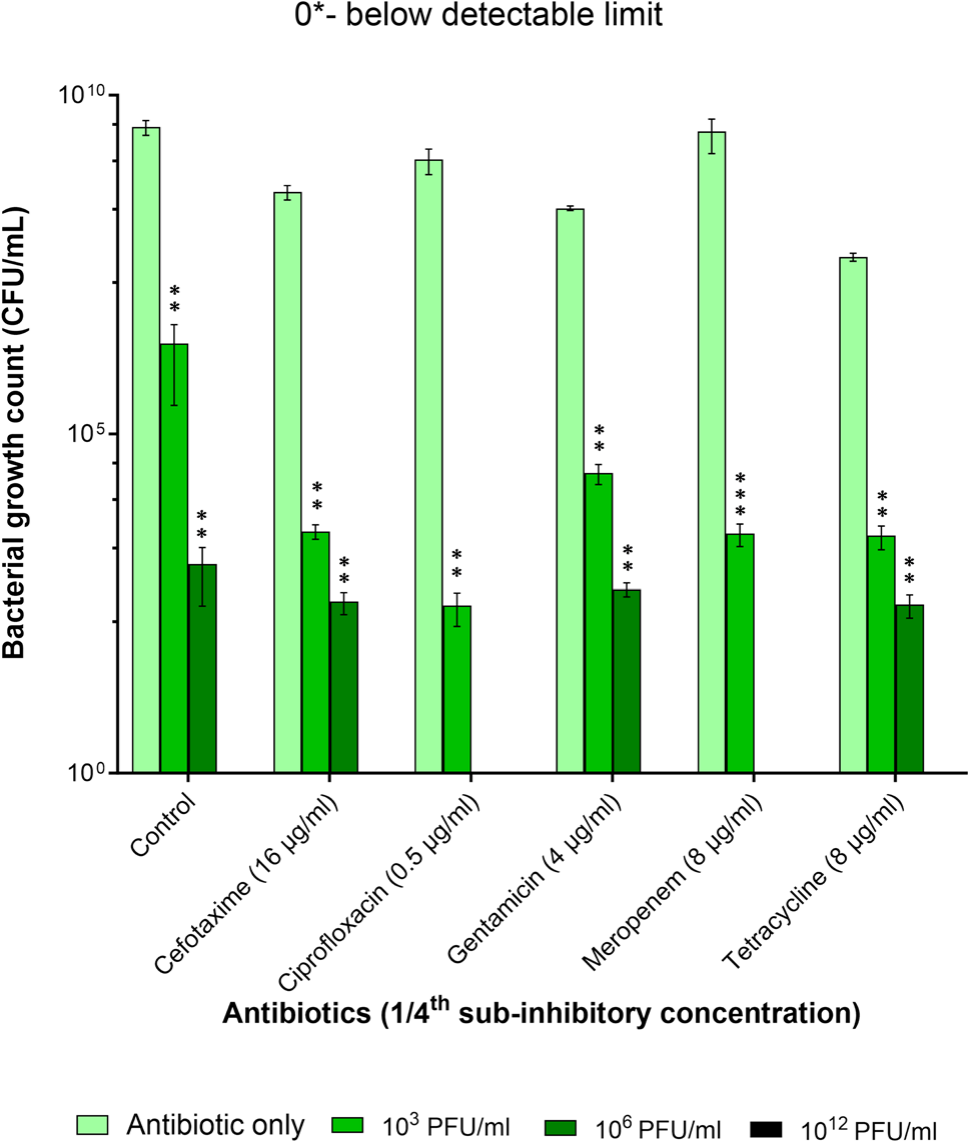
Synergistic effect of *Pseudomonas* phage Motto with antibiotics as determined by statistical analysis. The combination provides a significant reduction in bacterial growth as observed by CFU/mL. In case no bars are visible, the bacterial count was zero. All the error bars indicated are standard errors and the ***p < 0.001, **p > 0.05 were determined by two-way ANOVA (between antibiotic only and phage-antibiotic combination) with Bonferroni correction.

For the following experiments, sublethal concentrations of antibiotics were chosen and accordingly, 1/4th of the MIC was selected. Next, all the antibiotics were tested for their phage-inhibitory effects to ensure that the phage activity was not affected by the compounds (see Figure S1). The effect of *Pseudomonas* phage Motto and sublethal concentrations of the antibiotics on *P. aeruginosa* PA01 was then investigated by determining the viable cell concentration (CFU/mL) after 18 h of exposure. In the control group (without phage and antibiotic), the bacterial count was 3.4 × 10^9^ CFU/mL, whereas the phages with a final titer of 10^3^ and 10^6^ PFU/mL reduced the bacterial count to 2.1 × 10^6^ CFU/mL and 1.2 × 10^3^ CFU/mL, respectively (Fig. 1). No bacteria were found when the pathogen was exposed to phages at a titre of 10^12^ PFU/mL (Table 2).

**Table 2:** Combination of *Pseudomonas* phage Motto with antibiotics showed a synergistic effect.

Antibiotics (µg/mL)	Phage (PFU/mL)	Bacterial growth (CFU/mL)
Control (no antibiotic)	Control (no phage)	3.4 × 10^9^
Ciprofloxacin (0.5)	–	1.1 × 10^9^
Cefotaxime (16)	–	3.7 × 10^8^
Meropenem (8)	–	2.9 × 10^9^
Gentamicin (4)	–	2.1 × 10^8^
Tetracycline (8)	–	4.1 × 10^7^
–	10^3^	2.1 × 10^6^
–	10^6^	1.2 × 10^3^
–	10^12^	0*
Ciprofloxacin (0.5)	10^3^	3 × 10^2^
	10^6^	0*
	10^12^	0*
Cefotaxime (16)	10^3^	3.6 × 10^3^
	10^6^	3.4 × 10^2^
	10^12^	0*
Meropenem (8)	10^3^	3.4 × 10^3^
	10^6^	0*
	10^12^	0*
Gentamicin (4)	10^3^	2.6 × 10^4^
	10^6^	5.1 × 10^2^
	10^12^	0*
Tetracycline (8)	10^3^	3.1 × 10^3^
	10^6^	3.1 × 10^2^
	10^12^	0*

0*—below detectable limit.

In the absence of phages, antibiotics at sublethal concentrations (1/4th of the MIC) had minor or even no inhibitory effects. Accordingly, bacterial reductions were observed in the case of tetracycline (4.1 × 10^7^ CFU/mL), cefotaxime (3.7 × 10^8^ CFU/mL) and gentamicin (2.1 × 10^8^ CFU/mL) but no effect was found when ciprofloxacin was used (1.1 × 10^9^ CFU/mL). Likewise, meropenem had no significant effect (2.9 × 10^9^ CFU/mL). While we observed synergistic effects of phages with three antibiotics, ciprofloxacin, cefotaxime and meropenem. The combination of ciprofloxacin (0.5 µg/mL) together with phages at 10^3^ PFU/mL resulted in a seven-log reduction (Table 2). Similarly, meropenem (8 µg/mL) together with phages at 10^3^ PFU/mL resulted in a six-log reduction. Phages at a higher titre of 10^6^ PFU/mL (plus ciprofloxacin or meropenem) resulted in the complete elimination of bacterial cells. Cefotaxime-phage had synergistic activity resulting in a five-log (10^3^ PFU/mL) and six-log (10^6^ PFU/mL) reduction. The combination of 10^3^ PFU/mL phages together with gentamicin (4 µg/mL) resulted in a four-log reduction, and at 10^6^ PFU/mL a six-log reduction was observed. Tetracycline-phage combinations substantially reduced the number of viable cells: At a phage number of 10^3^ PFU/mL a four-log reduction was seen, while a higher titre of 10^6^ PFU/mL resulted in a five-log reduction of bacterial cells (Table 2).

### Phage-antibiotic combinations show synergy against biofilm-forming *P. aeruginosa* cells

Often phages and antibiotics have different effects on the same bacterial strain depending on whether they are embedded in a biofilm or planktonic^23,24^. Thus, we determined the possible effects of phage-antibiotic combinations on bacterial biofilm-embedded cells. After forming biofilm-embedded cells in microtiter plates for 24 h, we added different concentrations of antibiotics together with different amounts of phages. A synography model was previously designed by the TAILΦR lab in Texas, to study and visualise the effectiveness of phage-antibiotic combinations across many stoichiometries^4^.

**Figure 2:**
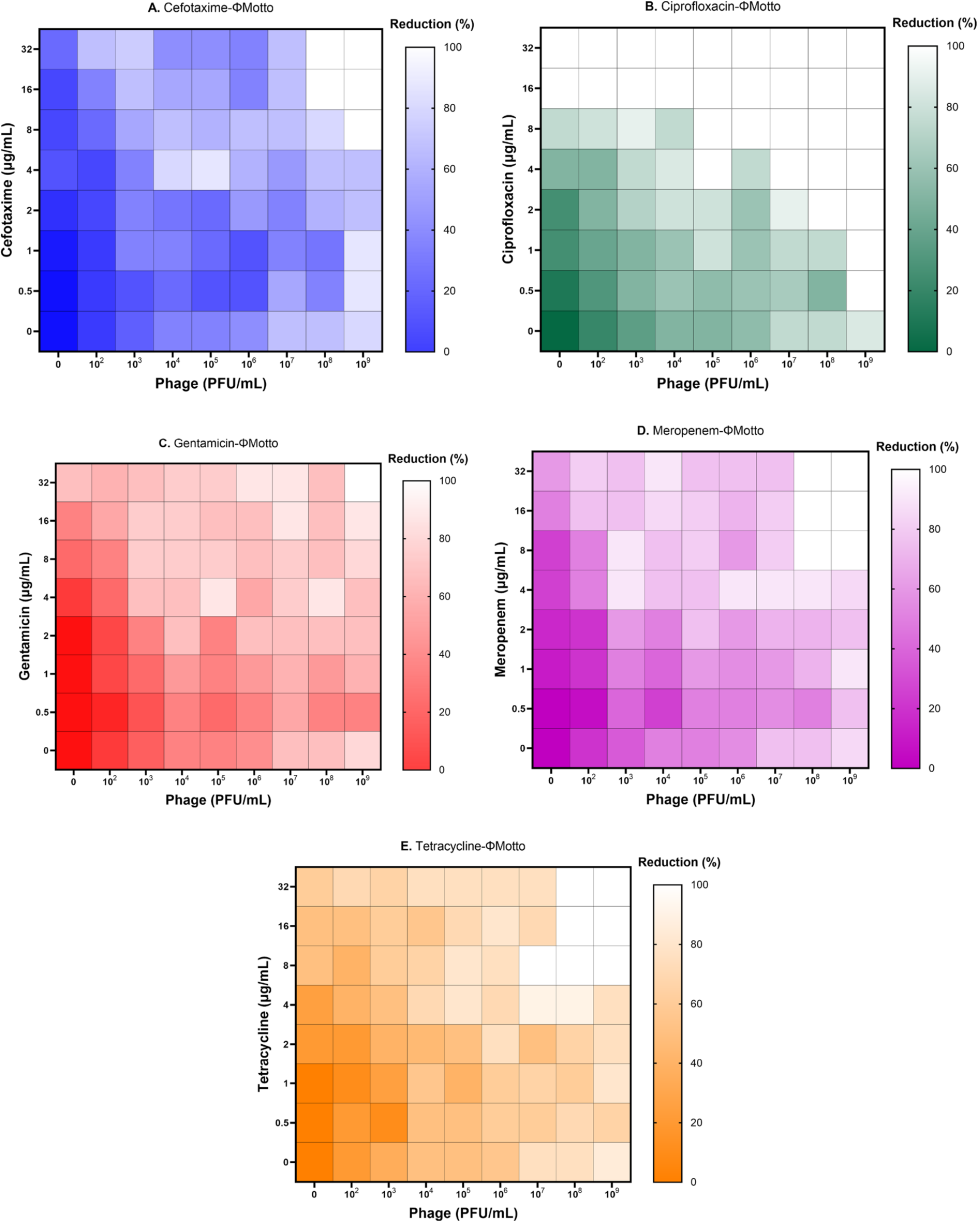
Anti-biofilm effect of *Pseudomonas* phage Motto and different antibiotics on *P. aeruginosa* biofilms. The effect of different antibiotics was studied: (**A**) Cefotaxime; (**B**) Ciprofloxacin; (**C**) Gentamicin; (**D**) Meropenem; (**E**) Tetracycline. The bacterial biofilms (24 h old) were treated with different combinations of phages and antibiotics. The synograms represent the OD_595nm_ values read after 24 h of treatment and the mean reduction percentage of each treatment from three independent replicates.

The analysis of our data shows that for all phage-antibiotic combinations, at high titres and antibiotic concentrations, the biofilm was reduced to a varying extent and depending on the antibiotic. In the treatment of the pseudomonal biofilm with cefotaxime alone, even the highest antibiotic concentration of 32 µg/mL did not show any significant anti-biofilm effect. However, complete removal of the biofilm was observed at concentrations of cefotaxime between 32 and 16 µg/mL when phages were present at high titres (Fig. 2A). MIC results showed that *P. aeruginosa* PA01 was sensitive to ciprofloxacin. Correlating with this, we observed a complete biofilm eradication at the higher concentrations of ciprofloxacin (> = 16 µg/mL) which appears at first glance surprising (Fig. 2B). Possibly the bactericidal effects lead to a release of bacterial enzymes from the cells which then degrade the biofilm as a direct effect of the ciprofloxacin is less likely; this interpretation however, is speculation and requires further experimental validation. Nevertheless, synergistic effects were observed with increasing amounts of phages. Gentamicin alone exhibited minor anti-biofilm properties but combinatorial effects can be seen when phages are added at titres higher than 10^3^ PFU/mL (Fig. 2C). Similarly, meropenem, which had no obvious effect on biofilms on its own, did only reduce the biofilm when phages were present. Clear synergy was observed at values higher than 4 µg/mL and titres higher than 10^7^ PFU/mL (Fig. 2D). While tetracycline alone showed -to some extent- anti-biofilm properties in our assay, the combination of the antibiotic with the phage exhibited significant synergistic effects at concentrations higher than 4 µg/mL and phage numbers higher than 10^7^ PFU/mL (Fig. 2E).

### Combinations of phage and the antifungal fluconazole have minor effects on dual-species biofilms

**Figure 3:**
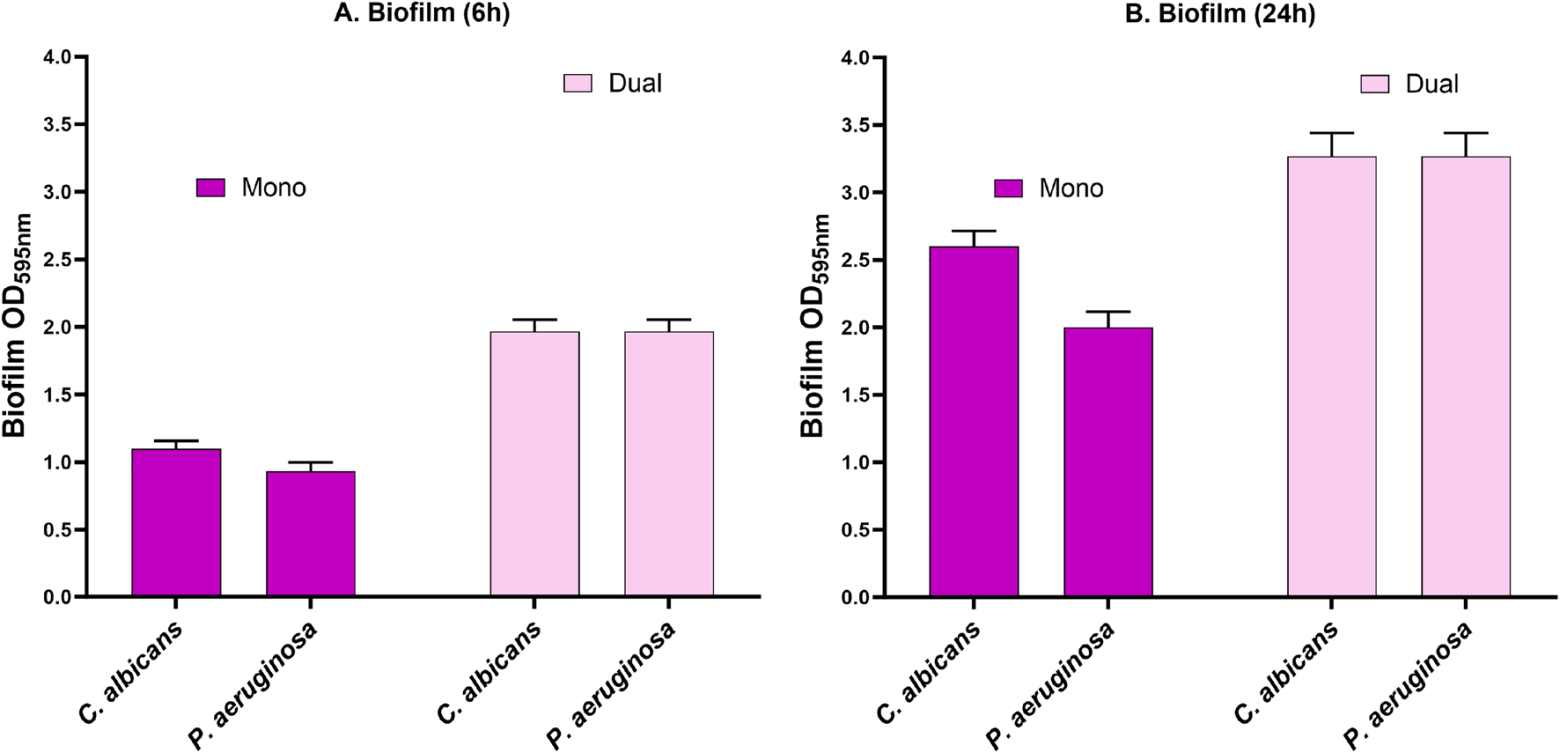
Biofilm of *C. albicans* and *P. aeruginosa* clinical isolates in mono- and dual-species at 6 h (left) and 24 h (right). The results represent the biofilm formation after 6 and 24 h as the absorbance was read at OD_595nm_ and the error bars represent the standard mean of the three independent experiments.

To investigate the formation of mono- and dual-species biofilms, we cultured cells of *P. aeruginosa* PA01 and *C. albicans* C11 either alone or together in microtiter plates. After 6 h, cultures of *P. aeruginosa* PA01 quantitatively formed less biofilm compared to *C. albicans* C11, according to the assay we employed. Both organisms together, however, formed even more biofilm than the individual ones combined (Fig. 3A). After 24 h, biofilms increased for both the bacterial and the fungal pathogen (mono), while the dual-species biofilm was less than the sum of the individual biofilms from *P. aeruginosa* PA01 and of *C. albicans* C11 (Fig. 3B).

**Figure 4:**
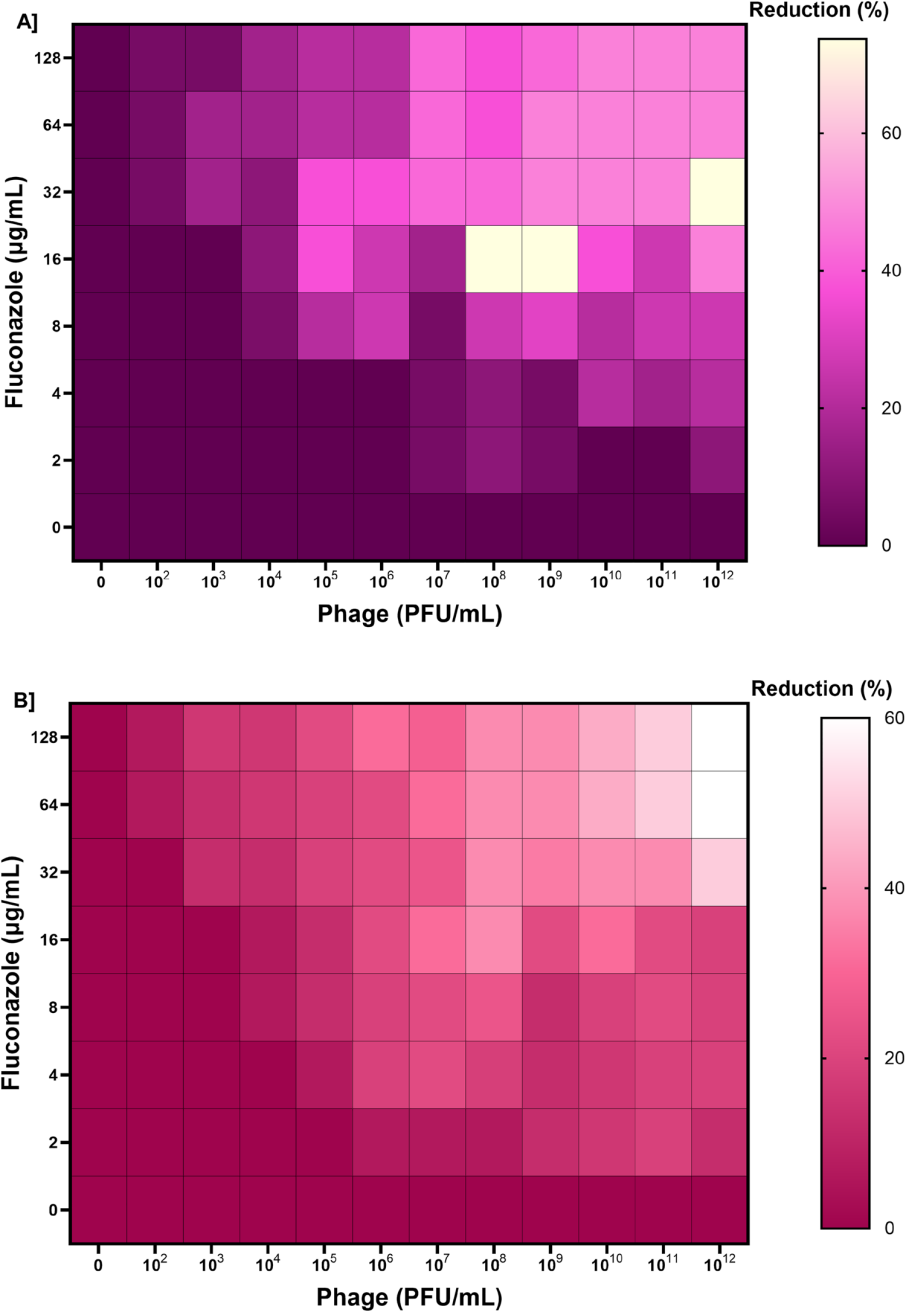
Phage-fluconazole synergism in the reduction of dual-species biofilms formed by *Pseudomonas*-*Candida*. Effect of *Pseudomonas* phage Motto (10^2^ to 10^12^) and fluconazole (128 to 2 µg/mL) on dual-species biofilms. The dual-species biofilms [6 h old (top) and 24 h old (bottom)] were treated with different combinations of *Pseudomonas* phage and fluconazole. The synograms represent the OD_595nm_ values as read after 24 h of treatment and the mean reduction percentage of treatment from three independent replicates.

The eradication of dual-species biofilms is impossible in the presence of phage alone or antibiotics alone as the fungal pathogen can multiply and continue to produce biofilm. Therefore, we tested phage-antifungal combinations. A complete eradication was not observed, even at the highest phage and fluconazole concentrations (Fig. 4). No synergistic effect was observed when a phage-antifungal combination was used against *C. albicans* (mono) biofilms (see Figure S2). Nonetheless, a trend at high phage and antibiotic concentrations with a biofilm-adverse effect can be observed in both cases of up to 30%, with 6 and 24-h biofilm samples (Fig. 4). Though crystal violet assay cannot distinguish between the two species in a biofilm, it is worth noting that at higher phage concentrations (without fluconazole) Motto was penetrating the *P. aeruginosa* biofilms. It is clear that the phage has a positive impact on the removal of the dual-species biofilms in combination with exposure to fluconazole.

### Antifungals are required for dual-species biofilm removal, as phage-antibacterial combinations have no effect

We established that phage-antibiotic combinations are effective on biofilms formed by *P. aeruginosa* alone. We also demonstrated that phage-antifungal combinations resulted in the reduction of biofilms formed by the bacterial pathogen together with *C. albicans*, albeit to a minor extent. Thus, we next tested if the combination of phages together with antibiotics acting on *P. aeruginosa* leads to a reduction of dual-species biofilms. However, even at the highest concentration and highest phage titre tested, biofilms remained unaltered (see Figure S3). This is somewhat surprising, as one might suspect that the bacterial biofilm is reduced. However, the fungal pathogen possibly continues to thrive and thus produces more biofilm. Crystal violet assay cannot distinguish between the two species in a biofilm. It is possible that the phage-antibiotics are killing *P. aeruginosa* but not *C. albicans*, allowing *C. albicans* to grow. If *P. aeruginosa* is being killed, another explanation is the size difference between the two species, causing crystal violet to stain *C. albicans* primarily. Alternatively, it is possible that the presence of *C. albicans* and its extracellular matrix components protects *P. aeruginosa* from the phage-antibiotic treatment.

### Antimicrobial combinations of the antifungal fluconazole together with antibacterials act synergistically at high concentrations

**Figure 5:**
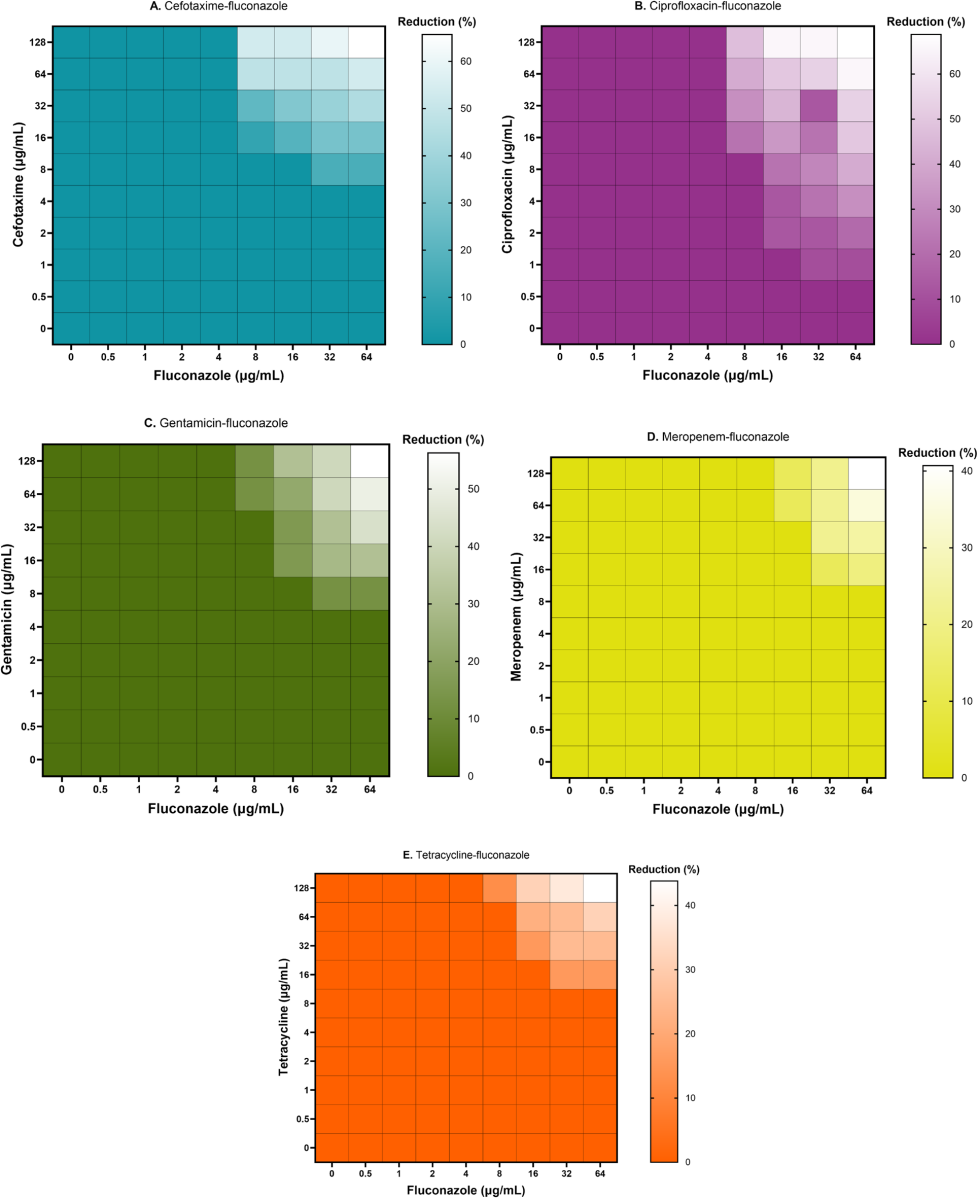
Fluconazole-antibiotic synergism in the reduction of dual-species biofilms formed by *Pseudomonas*-*Candida*. Effect of fluconazole (0.5–64 µg/mL) and antibiotics (cefotaxime, ciprofloxacin, gentamicin, meropenem, tetracycline at 0.5–128 µg/mL) on dual-species biofilms. The dual-species biofilms (24 h old) were treated with different combinations of fluconazole and antibiotics. The synograms represent the OD_595nm_ values as read after 24 h of treatment and the mean reduction percentage of treatment from three independent replicates.

Although this study focused on the antibiofilm properties of a *P. aeruginosa* phage in combination with antimicrobial compounds, we also investigated how antibiotics alone act on dual-species biofilms. Interestingly, we observe antibiotic-specific synergy in some cases; clear effects can be seen in the case of cefotaxime and ciprofloxacin up to 60% reduction, while the reduction of biofilm using gentamicin together with fluconazole was less pronounced with a 50% reduction. Both meropenem and tetracycline in conjunction with the antifungal were unable to reduce the biofilm with the reduction of < 40% (Fig. 5).

## Discussion

*Pseudomonas aeruginosa* is known to cause life-threatening infections in humans and is one of the common nosocomial pathogens to cause respiratory tract infections. To overcome the threat of antibiotic-resistant pseudomonal infections, non-antibiotic therapies and antibiotic combinatorial therapies are being investigated as valuable alternatives^25^. The use of phage-antibiotic combination therapies has been therapeutically successful, possibly due to the emergence of phage-resistant strains that show increased antibiotic susceptibility^8,26,27^. However, many infections are complicated by the formation of biofilms, which inhibit the diffusion of antibiotic compounds and form a kind of protective layer around the pathogens. Due to their unique properties, phages have been found to be effective in dissolving biofilms, both in vitro and in vivo^28,29^. This was also demonstrated in the case of *P. aeruginosa* infections^30,31^. Thus, phages can act synergistically as a biofilm-disrupting agent, allowing antibiotics to penetrate the target. Despite this, many infections are caused not by one species but by several, known as polymicrobial infections. Such “collaborative infections” are common for *P. aeruginosa* which is often found together with *S. aureus*^32^. Especially in the lungs of cystic fibrosis (CF) patients, *P. aeruginosa* is present with *C. albicans*^33,34^. Thus, there is growing interest in multispecies biofilms. This is particularly important for those that are formed by different domains of life. The treatment failures are mainly due to the impermeability of dual-species biofilms by chemotherapeutic drugs.

Our study aimed to investigate the potential of phage-antibiotic combinations in eradicating biofilms formed by *P. aeruginosa* and dual-species biofilms of *P. aeruginosa*-*C. albicans*. Initially, *P. aeruginosa* PA01 planktonic cells were treated with phage-antibiotic combinations in order to establish a baseline for potentially observed phage-antibiotic synergy. Here, we found that ciprofloxacin and meropenem had a more pronounced synergistic effect (Fig. 1). On the other end of the spectrum, cefotaxime, gentamicin, and tetracycline had the least degree of synergism. Despite the bacteria being susceptible to ciprofloxacin (MIC of 0.5 µg/mL), when combined with phages even at the sublethal concentrations (1/4th MIC), a lethal anti-pseudomonal effect was observed (Fig. 1). The three antibiotics (ciprofloxacin, cefotaxime and meropenem) had a synergistic effect with a minimum of four-log reductions when compared to antibiotics alone and at least two-log reductions when compared to phages alone. Notably, irrespective of the antibiotic class, the *Pseudomonas* phage Motto was found to work synergistically with all the tested antibiotics in this study (Fig. 1). Our data is similar to a recent study which showed the anti-pseudomonal effect of phage-antibiotic (sub-MIC) combinations on pathogenic *P. aeruginosa* strains^27^. As we were interested in the effects on biofilm, we tested the reduction of pseudomonal biofilms when exposed to phage-antibiotic combinations (Fig. 2). Here, we observed that phage-ciprofloxacin exhibited enhanced biofilm eradication compared to other antibiotics (cefotaxime, gentamicin, meropenem and tetracycline) (Fig. 2). Similar results were reported previously showing that the phage-ciprofloxacin combination has better biofilm eradication ability compared to the single-compound treatment^14^. In our study, the synogram showed that phage Motto was acting synergistically with all tested antibiotics and was effective at disrupting *P. aeruginosa* biofilms (Fig. 2).

As *P. aeruginosa* is often found together with *C. albicans* in biofilms, we focused on this important aspect next: The inhibition of *P. aeruginosa*-*C. albicans* dual-species biofilms when exposed to the combination of phage together with the fungicide fluconazole were tested. To this end, we investigated the formation of biofilm by the pathogens on their own or when grown together. Curiously, while *P. aeruginosa* PA01 and *C. albicans* C11 were forming moderate amounts of biofilm within 6 h, we observed that the production of biofilm was additive when both pathogens were present. More substantial biofilms were produced by the individual species after 24 h, while the dual-species biofilm reached even higher quantities in our assay, yet not the sum of the individual amounts of mono-species biofilms (Fig. 3).

After establishing the extent of the formation of biofilms at the two-time points, we next tested the combination of the phage Motto together with the antifungal compound fluconazole. While complete biofilm eradication was observed in the case of 6-h-old biofilms, the 24-h-old biofilms were only partially (50%) disintegrated after 16 h of phage-fluconazole exposure (Fig. 4). A possible explanation for this strong difference is the change in biofilm composition over time, which remains to be investigated. While many phages are known to degrade bacterial biofilms, whether the depolymerases produced by phages can degrade dual-species biofilms, especially the ones made by fungi, remains unknown.

We also tested the combination of antibacterial compounds together with the antifungal drug (Fig. 5). Here, we observed a clear synergy albeit at high concentrations of both compounds. To our surprise, no effects (overall biomass) were seen at all when phage-antibacterial combinations were used to treat the dual-species biofilm (see Figure S3). A previous study had also showed meropenem tolerance of *P. aeruginosa* in the presence of *C. albicans* in dual-species biofilms^35^. However, one other study conducted previously showed the effect of phage combinations and ciprofloxacin to limit *S. aureus*- *C. albicans* biofilms^36^. Our study aimed to shed light on the complexity of polymicrobial biofilms and the effects of phages on their structure. At this point, there is no concluding evidence to prove the eradication of polymicrobial infections caused by pathogenic bacteria and fungi using phage-antibiotic combinations. It becomes obvious that we need more studies to understand the effect of phages on such complex systems in order to develop treatment regimes for dual-species infections or biofilm removal before phages can be considered for therapeutic purposes.

### Limitations of the study

Our study clearly illustrates the potential of using phages to disintegrate *P. aeruginosa* biofilms. Anti-biofilm properties are more pronounced when antibiotics are used together with the phage. The effect is not an additive one, as the compounds together exhibit synergy. However, underlying mechanisms for this synergy remain speculative as antibiotics on their own do not have anti-biofilm properties. Possibly, a re-sensitisation to antibiotics occurs in bacteria that acquire phage resistance, in turn leading to bacterial killing by the antibacterial compounds, as was previously observed^7,8,37^. When studying dual-species biofilms, the composition of the material is likely to be highly heterogeneous, as they are produced by both organisms. Such biofilms might possibly be even different from the combination of those formed by each individual species alone; there might be a yet unknown interplay, influencing the structure and composition of the matrix when both opportunistic pathogens are present. Using phages for the treatment of infections caused by two species might lead to *C. albicans* producing more biofilm and replacing the bacterial one. The fungal biofilm is unlikely to be efficiently degraded by the phage-derived enzymes that are known to often show specificity towards certain bacteria.

## Materials and methods

### Bacteria, Candida and bacteriophages

In this study, pathogenic mucoid *P. aeruginosa* PA01 and *C. albicans* C11 were used which were previously collected by the diagnostic centres in Chennai, Tamil Nadu, India. *C. albicans* C11 was isolated from the urine sample which is associated with virulence. Minimal inhibitory concentration (MIC) tests were performed following the micro-broth dilution method using cefotaxime, ciprofloxacin, gentamicin, meropenem and tetracycline (Merck KGaA, Darmstadt, Germany). The results were interpreted according to CLSI guidelines. Previously characterized lytic *Pseudomonas* phage Motto was used in this study^21,22^.

### Phage-antibiotic combination against planktonic *P. aeruginosa*

To study synergistic effects, the antibiotics were added at the sub-inhibitory concentrations (1/4th MIC) and the phages were used at concentrations of 10^3^, 10^6^ and 10^12^ PFU/mL. Briefly, antibiotics were diluted to the sub-inhibitory concentrations (1/4th MIC) which were mixed with the 50 µL of phages at respective concentrations in the micro-titre plates. Then, 5 µL of *P. aeruginosa* at 5 × 10^5^ CFU/mL was added. The plates were incubated at 37 °C for 18 h and 100 µL was removed, serially diluted (up to 10^–6^) and plated on LB agar plates. Colony-forming units (CFU/mL) were determined and compared to the control (phage and antibiotic alone). All the data are presented as mean ± standard deviation (SD) of at least three independent experiments.

### Phage-antibiotic combination against biofilm-forming *P. aeruginosa*

For the anti-biofilm studies, cefotaxime, ciprofloxacin, gentamicin, meropenem and tetracycline were used. Briefly, biofilms were formed using the exponentially growing bacterial cells from the overnight culture. Overnight cultures were grown at 37 °C in LB broth and diluted in a microtiter plate and incubated at 37 °C for 24 h. The phage-antibiotic combinations were prepared following the checkerboard assay (antibiotics from 0.5 to 32 µg/mL were prepared in columns and the phage concentrations from 10^2^ to 10^9^ PFU/mL were prepared in rows; the study design is represented in Figure S4). Then, the plate was incubated at 37 °C for 24 h, washed twice to remove the planktonic cells then stained using crystal violet. The optical density (OD) was determined at 595 nm using a microtitre plate reader (BioTek, India).

### Phage-antibiotic effects on dual-species biofilms

To study the inhibitory effect of *Pseudomonas* phage Motto and antimicrobial compounds including the fungicide fluconazole against *P. aeruginosa*-*C. albicans* mixed species biofilms, we allowed a dual-species biofilm to be formed: Briefly, overnight cultures of *P. aeruginosa* PA01 were grown at 37 °C in LB broth (tryptone, yeast extract and NaCl), and overnight cultures of *C. albicans* C11 were grown at 30 °C with shaking in YPD (Yeast extract, Peptone, and Dextrose) medium. For dual-species biofilms, equal volumes (equivalent to an OD_600_ value of 0.1) of *P. aeruginosa* and *C. albicans* (prepared in their respective medium) were mixed and incubated at 37 °C for 6 and 24 h. Plates were then washed twice to remove the planktonic cells. Again, checkerboard assays were performed as represented in Figure S1. Briefly, phages at 10^2^ to 10^12^ PFU/mL and fluconazole from 2 to 128 µg/mL were added (fluconazole in columns and phages in rows) and transferred to dual-species biofilm plates either after 6 h or 24 h (96-well microtiter plates). After the addition of phage-fluconazole combinations, the plates were incubated for 16 h, washed, and stained using crystal violet and OD_595nm_ was measured. The control groups include pseudomonal biofilm without challenge, *C. albicans* biofilm without challenge, and dual-species biofilm challenged with phage alone and fluconazole alone. All the data are presented as mean ± standard deviation (SD) of at least three independent experiments.

In the case of phage-antibacterial agents, the dual-species biofilms (24 h old) were treated with phages at 10^2^ to 10^9^ PFU/mL and antibacterials (cefotaxime, ciprofloxacin, gentamicin, meropenem and tetracycline) at 0.5–128 µg/mL. Accordingly, the dual-species biofilms (24 h old) were treated with fluconazole at 0.5 to 64 µg/mL and antibacterials (cefotaxime, ciprofloxacin, gentamicin, meropenem and tetracycline) at 0.5 to 128 µg/mL. After the addition of phage/fluconazole-antibacterial combinations, the plates were incubated for 16 h, washed then stained using crystal violet and OD_595nm_ was measured. Statistical analysis was performed using GraphPad Prism software 7.0 (GraphPad Software, Inc., La Jolla, USA).

### Supplementary Information

Supplementary Figure S1.

Supplementary Figure S2.

Supplementary Figure S3.

Supplementary Figure S4.

## Data Availability

Genome sequence data have been deposited at NCBI and are publicly available as of the date of publication. The accession number of the *Pseudomonas* phage Motto is ON843697. Any additional information required to reanalyze the data reported in this paper is available from the lead contact upon request.
