# Supplementary material for: Phage-antibiotic combinations to control Pseudomonas aeruginosa–Candida two-species biofilms
Source: Sci Rep. 2024 Apr 23;14:9354. doi: 10.1038/s41598-024-59444-2 (PMC11039464; doi:10.1038/s41598-024-59444-2)
Supplement: Supplementary file 1 — Supplementary Figure S1. [file 41598_2024_59444_MOESM1_ESM.docx]

**Supplemental materials:**

**Supplemental figures:**


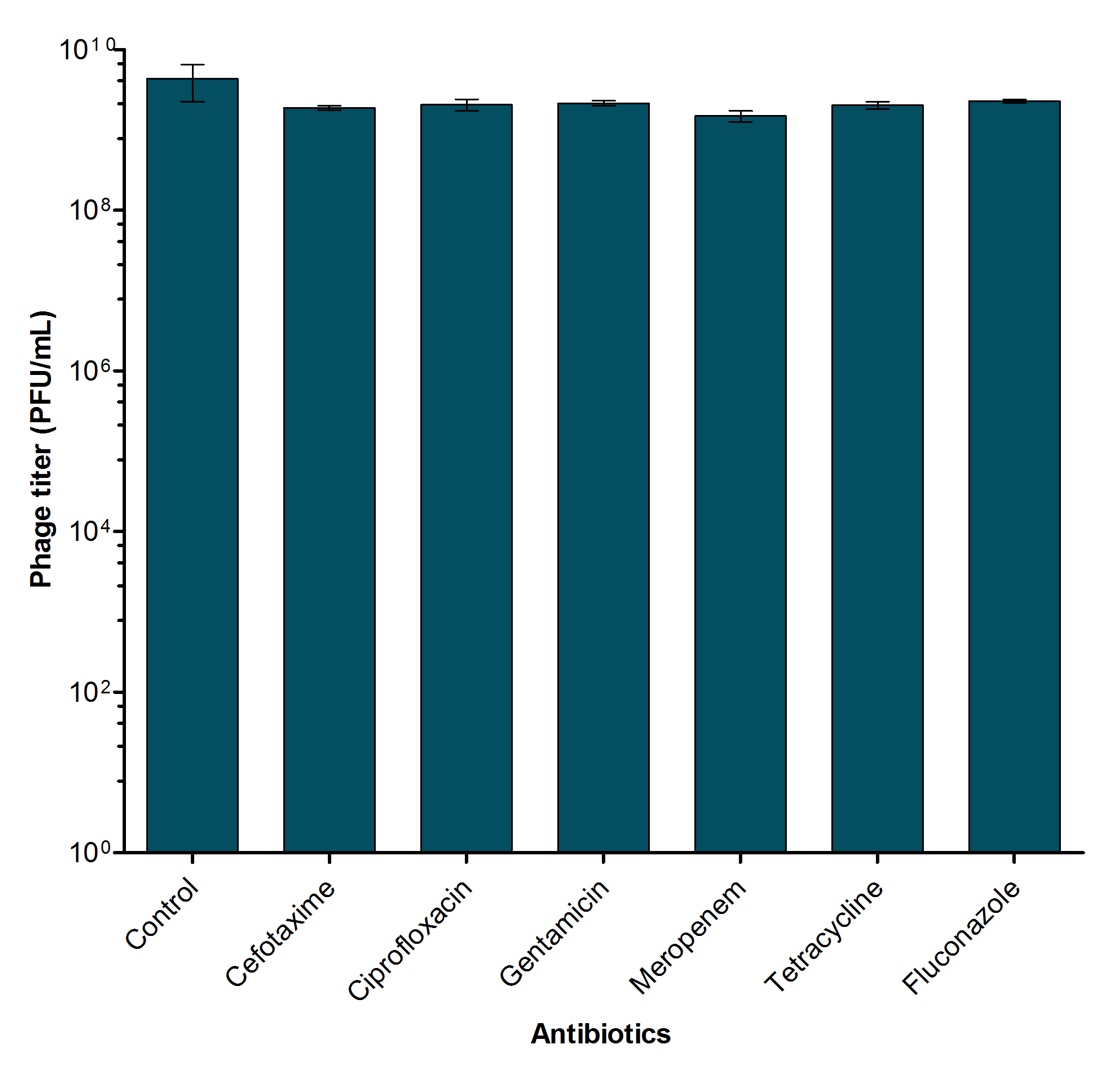


**Figure S1: Phage titer after treatment with different antibiotics.**
