# Supplementary material for: Phage-antibiotic combinations to control Pseudomonas aeruginosa–Candida two-species biofilms
Source: Sci Rep. 2024 Apr 23;14:9354. doi: 10.1038/s41598-024-59444-2 (PMC11039464; doi:10.1038/s41598-024-59444-2)
Supplement: Supplementary file 2 — Supplementary Figure S2. [file 41598_2024_59444_MOESM2_ESM.docx]

**Supplemental materials:**

**Supplemental figures:**


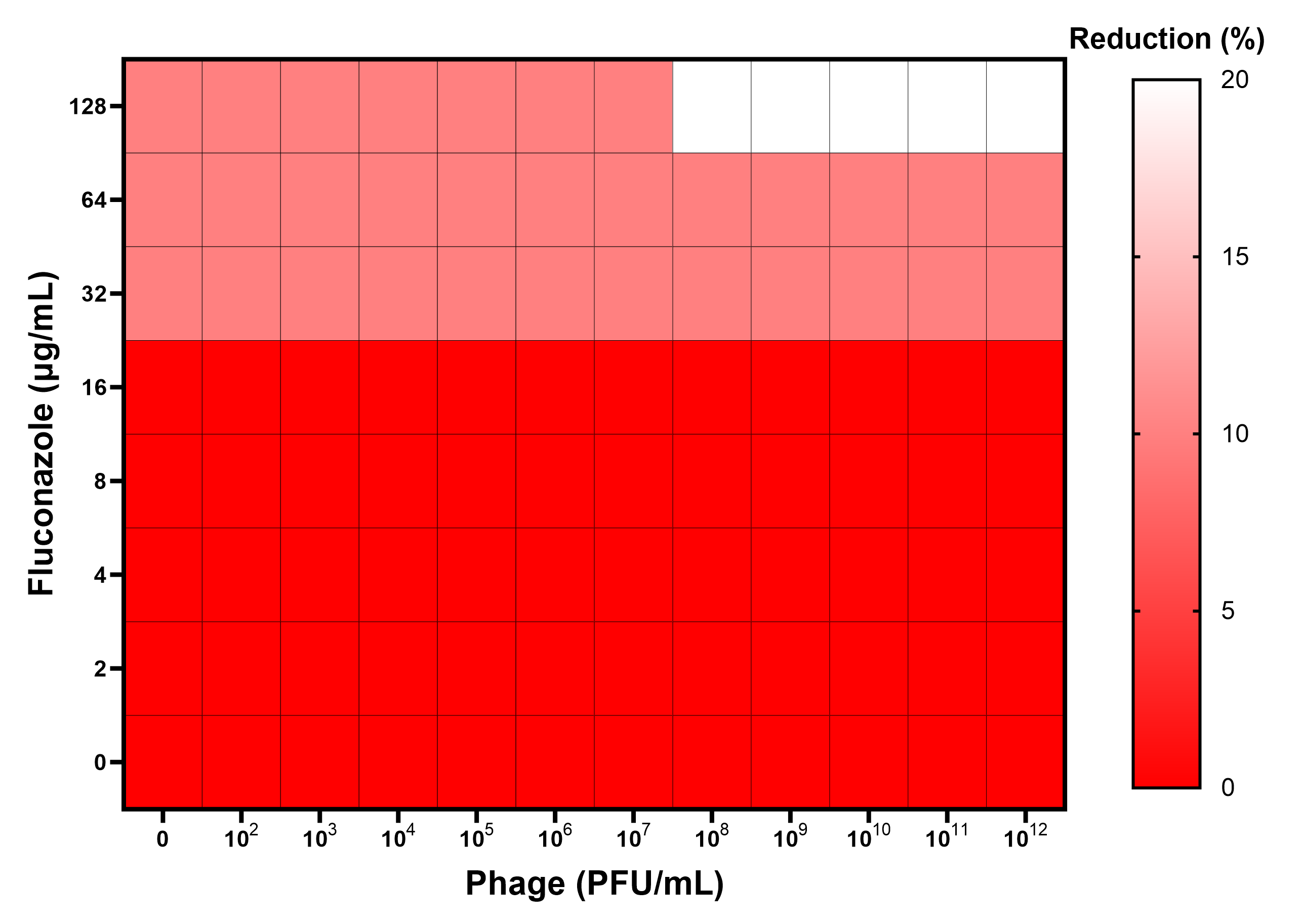


**Figure S2:** **Phage-fluconazole combination against mono-species biofilms formed by *Candida albicans*.** Effect of *Pseudomonas* phage Motto (10^2^ to 10^12^) and fluconazole (128 to 2 µg/mL) on mono-species biofilms. The mono-species biofilms [24 hours old] were treated with different combinations of *Pseudomonas* phage and fluconazole. The synograms represent the OD_595nm_ values as read after 24 hours of treatment and the mean reduction percentage of treatment from three independent replicates.
