# Supplementary material for: Phage-antibiotic combinations to control Pseudomonas aeruginosa–Candida two-species biofilms
Source: Sci Rep. 2024 Apr 23;14:9354. doi: 10.1038/s41598-024-59444-2 (PMC11039464; doi:10.1038/s41598-024-59444-2)
Supplement: Supplementary file 3 — Supplementary Figure S3. [file 41598_2024_59444_MOESM3_ESM.docx]

**Supplemental materials:**

**Supplemental figures:**

**
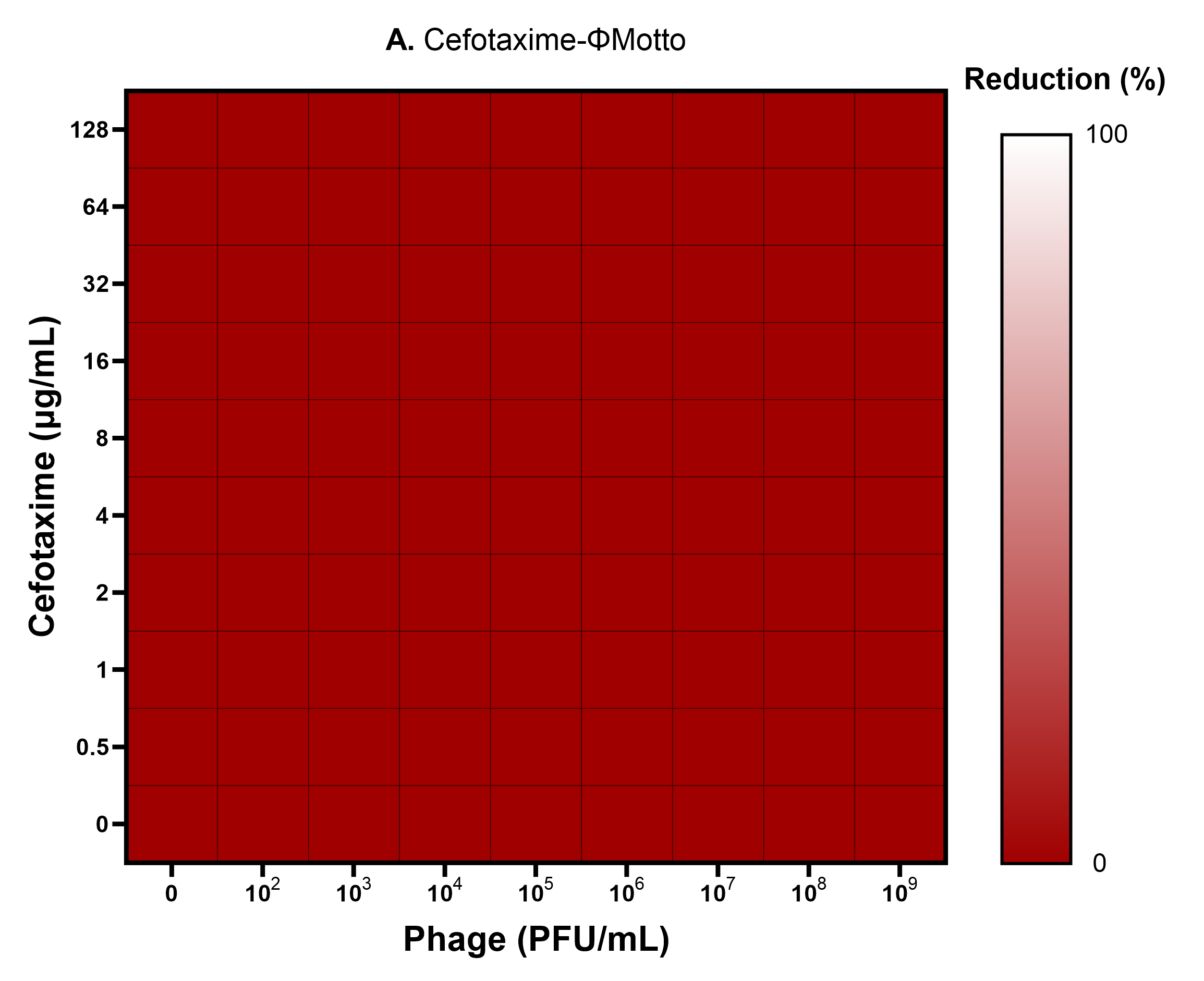

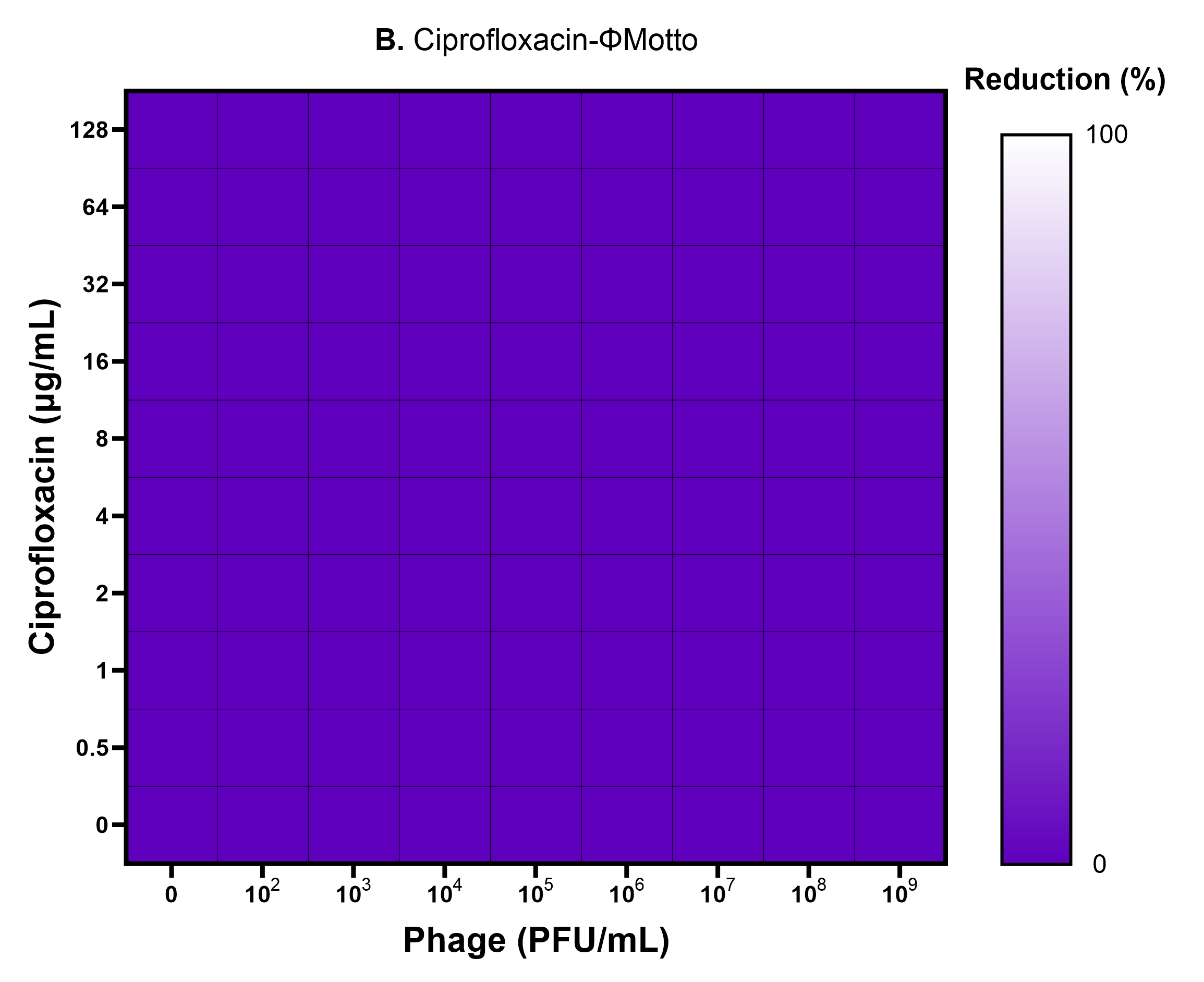
**

**
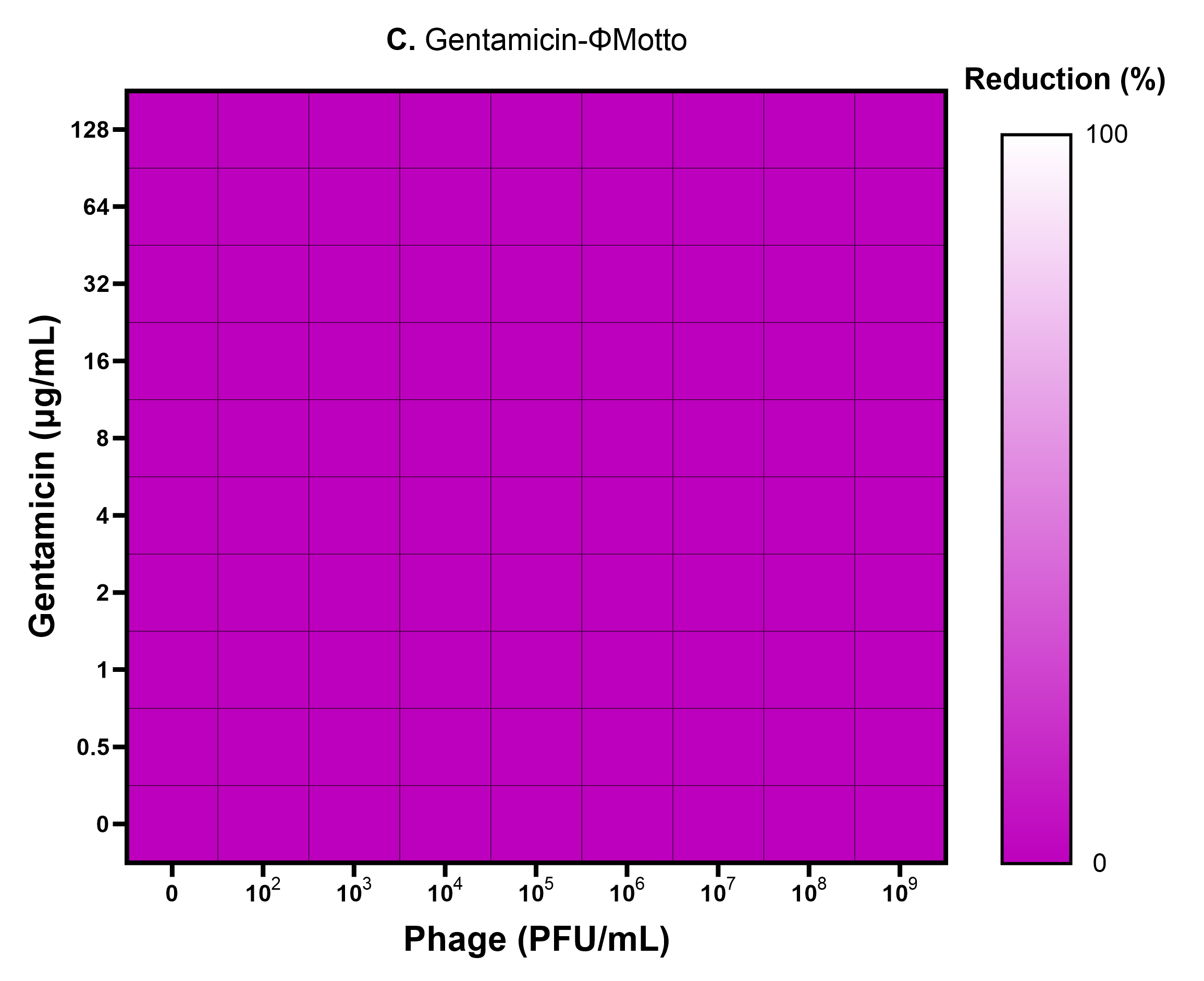

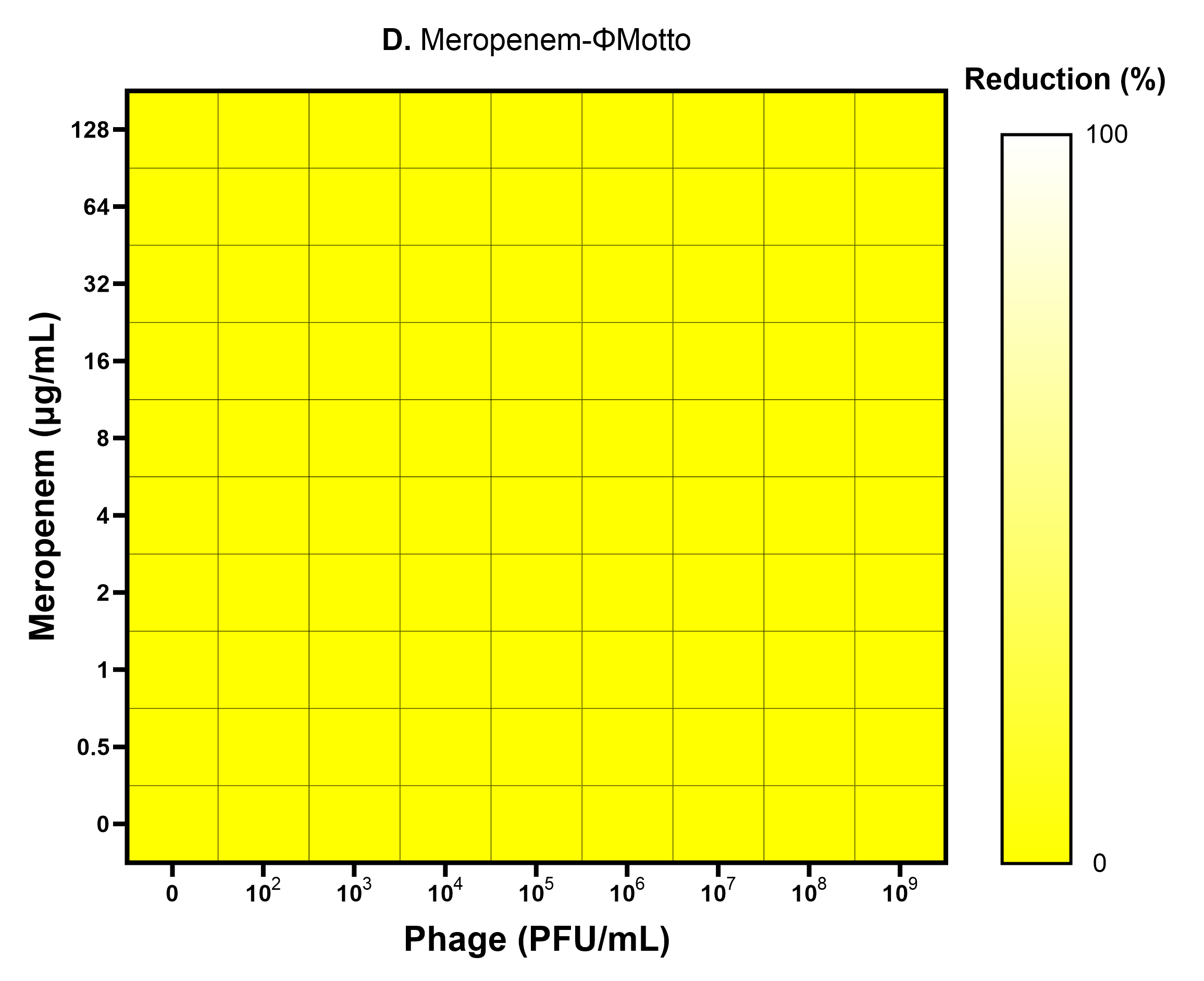
**

**
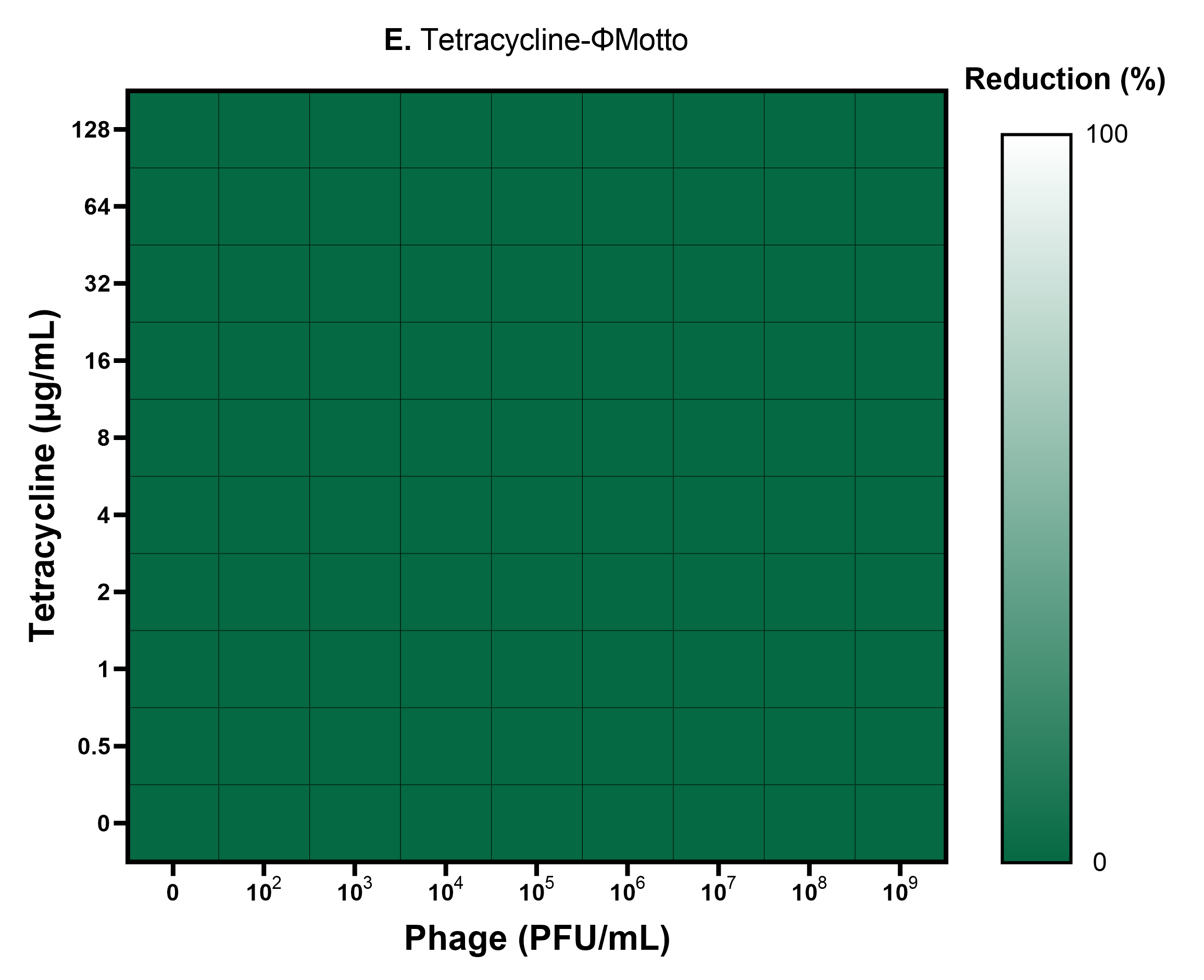
**

**Figure S3: Phage-antibiotic synergism in the reduction of dual-species biofilms formed by *Pseudomonas*-*Candida*.** Effect of *Pseudomonas* phage Motto (10^2^ to 10^9^) and antibiotics (cefotaxime, ciprofloxacin, gentamicin, meropenem, tetracycline) on dual-species biofilms. The dual-species biofilms (24 hours old) were treated with different combinations of *Pseudomonas* phage and antibiotics. The synograms represent the OD_595nm_ values as read after 24 hours of treatment and the mean reduction percentage of treatment from three independent replicates.
