# Supplementary material for: Phage-antibiotic combinations to control Pseudomonas aeruginosa–Candida two-species biofilms
Source: Sci Rep. 2024 Apr 23;14:9354. doi: 10.1038/s41598-024-59444-2 (PMC11039464; doi:10.1038/s41598-024-59444-2)
Supplement: Supplementary file 4 — Supplementary Figure S4. [file 41598_2024_59444_MOESM4_ESM.docx]

**Supplemental materials:**

**Supplemental figures:**

**
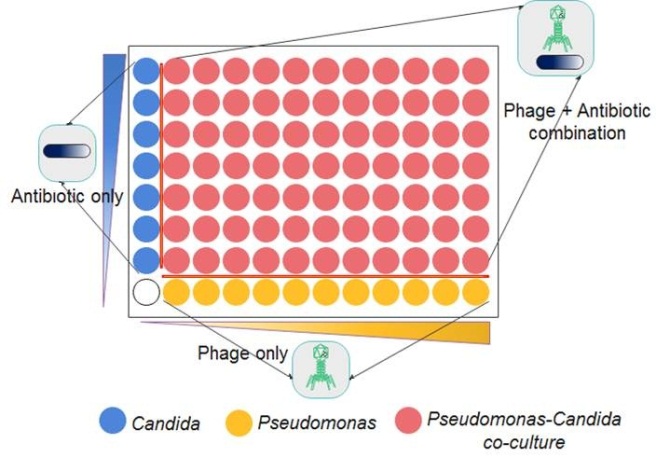
**

**Figure S4: Illustration of combination experiments performed in this study.** The dilution was performed using micro-titre plates and the varying concentrations of phages from 10^2^ to 10^9^ PFU/mL or fluconazole from 0.5 to 32 µg/mL or antibacterials from 0.5 to 128 µg/mL were prepared as depicted. **(1)** In the case of *Pseudomonas* phage Motto and fluconazole combination, the dilution was performed at varying concentrations of phages from 10^2^ to 10^12^ PFU/mL (bottom to top) and fluconazole from 2 to 128 µg/mL (left to right) was prepared. **(2)** In the case of *Pseudomonas* phage Motto and antibacterial combination, the dilution was performed at varying concentrations of phages from 10^2^ to 10^9^ PFU/mL (bottom to top) and antibacterials from 2 to 128 µg/mL (left to right) were prepared. **(3)** In the case of fluconazole and antibacterial combination, the dilution was performed at varying concentrations of fluconazole from 0.5 to 64 µg/mL (bottom to top) and antibacterials from 2 to 128 µg/mL (left to right) were prepared.
